# Improving Worst-Case Delay Analysis for Traffic of Additional Stream Reservation Class in Ethernet-AVB Network

**DOI:** 10.3390/s18113849

**Published:** 2018-11-09

**Authors:** Lin Zhao, Feng He, Ershuai Li, Huagang Xiong

**Affiliations:** School of Electronic and Information Engineering, Beihang University, Beijing 100191, China; zhaolin@buaa.edu.cn (L.Z.); Ershuai_li@hotmail.com (E.L.); hgxiong@buaa.edu.cn (H.X.)

**Keywords:** automotive, credit-based shaper, deterministic delay analysis, Ethernet-AVB

## Abstract

With the increase in the number of Electronic Control Units (ECUs) and future requirements for vehicle functions, two SR (Stream Reservation) traffic classes may not be sufficient to ensure fulfilment of constraints for multiple traffic types with individual timing requirements transmitted in the Ethernet-AVB (Audio Video Bridging) networks. The goal of this paper is to determine the worst-case delay for an additional SR traffic class under the CBS (Credit-Based Shaper) algorithm. Delay evaluation is based on the impact analysis of CBS on different priority flows, particularly depending on when the credits of both SR class A and B drain from the worst-case perspective. More specifically, both the impact of CBS and the evolution trends of credit on different priority class flows are first analyzed from the worst-case perspective. Then, for an additional SR class, two types of worst-case delay models are established with the CBS configuration suggestions. Finally, an approach to calculate the worst-case queuing delay is proposed. Moreover, the worst-case end-to-end delay is determined by the network calculus approach and simulation. Numerical results show that the delay bounds of our models are tighter than those of other models, which is beneficial to the development of Ethernet-AVB for in-vehicle networking.

## 1. Introduction

Owing to the open standard, high bandwidth, simplicity and low cost characteristics, Ethernet-based networking solutions are most promising for vehicle networks [1]. Several solutions have been presented, including Avionics Full-Duplex Switched Ethernet (AFDX), Time-Triggered Ethernet (TTEthernet) and IEEE Ethernet-AVB (Audio Video Bridging). Thanks to the Virtual Link (VL) concept, bandwidth reservation strategy and redundancy mechanism, AFDX (ARINC 664-part 7 [2]) has been successfully used in civilian aircrafts such as the Boeing 787 and Airbus A380. An improvement on AFDX, TTEthernet (SAE AS6802 [3]) satisfies strict timing transmission by use of a mixture infrastructure to support Time-Triggered (TT), Rate-Constraint (RC) and Best-Effort (BE) traffics. The key feature of TTEthernet is a time-triggered communication paradigm according to the off-line schedule table based on global synchronization [4]. IEEE 802.1 introduces guaranteed timing behavior with the focus on transportation of audio and video streams. IEEE Ethernet-AVB [5,6,7,8] is designed as a real-time communication network for multimedia streams with low delay and low jitter. It adopts a Credit-Based Shaper (CBS) on top of the Strict Priority Queuing (SPQ) forwarding policy. Ethernet-AVB’s further proposal TSN (Time Sensitive Networking) [9] develops new shaping mechanisms for Control Data Traffic (CDT) to support hard real-time applications, but some TSN standards are still in progress.

### 1.1. Related Work

Much research concerns performance comparisons of the above technologies [10,11,12]. In particular, previous work [10] aims at giving a comprehensive overview of the basic principles behind Ethernet-AVB and mapping them to AFDX. In ref. [11], AFDX, TTEthernet, and Ethernet-AVB are compared in terms of cost, the physical layer, topology, redundancy, security, etc. In ref. [12], the authors present the difference between unsynchronized and synchronized communication and highlight the trends and new horizons in real-time network configurations. Among these technologies, Ethernet-AVB as well as TSN shows great interest as it provides dedicated credit-based bandwidth for multimedia flows relying on a CBS algorithm to satisfy the transmission requirements of the automotive industry [13,14,15,16]. The work [17] suggests that the scheduling architecture of Ethernet-AVB should be a candidate for the evolution of AFDX including BE flows. In ref. [18], the authors indicate that CBS could bring additional delays for the highest priority flows during the corresponding credit recovery interval but potentially provides a fairness condition for low priority flows forwarded in switches. Bordoloi et al. [19] builds a necessary condition for the schedulability of class A and B at an output port supporting the CBS algorithm.

In the context of time-critical systems, the challenge lies in the calculation of worst-case delay. Some network solutions, such as Ethernet-AVB and TTEthernet, are compared in terms of end-to-end delay [20,21]. The worst-case end-to-end delay for one frame of a flow transmitted over a network can be broken out into two fixed parts (transmission delay, switching delay) and one variable part (queuing delay) [22]. The transmission delay over a link depends on the frame length and link bandwidth. Switching delay is the delay in switches between input and output ports. It is a constant value with an upper bound of 16 μs. Queuing delay depends highly on each output port load at the time when the considered frame reaches it. It is not deterministic and lies in the defined scenario.

Existing approaches for the worst-case delay analysis are divided into three categories [23]: Model-checking approach, simulation approach and some mathematical analysis approaches. The model-checking [24] approach depends on an exhaustive exploration of all possible scenarios to obtain an exact worst-case end-to-end delay. Unfortunately, this approach cannot cope with large-scale industrial configurations due to the combinatorial explosion problem. The simulation approach achieves the maximum delay observed on a set of scenarios but cannot provide guarantees on worst-case end-to-end delay. Some mathematical analysis approaches compute a sure upper-bound delay with a certain pessimism, such as network calculus [25,26], the trajectory approach [27] and the holistic approach [28]. In particular, network calculus is a mature approach that has been successfully used for worst-case delay analysis in the AFDX network [29,30]. In refs. [31,32], the authors discuss Ethernet-AVB network calculus models. In refs. [33,34], network calculus is used to make timing analysis of AVB traffic in TSN networks. In ref. [35], an improved trajectory approach is considered to obtain tight flow delay bounds with serialization constraints in the Ethernet-AVB network.

### 1.2. Motivation and Contributions

According to 802.1Q [36], each Ethernet-AVB output port offers up to 8 traffic classes including a maximum of two SR traffic classes A and B with highest priority (A having higher priority than B) plus six non-SR traffic classes, such as Best Effort (BE) traffic. In a current premium car, there are up to 70 Electronic Control Units (ECUs) with more than 900 functions interconnected over the in-vehicle network [21]. Each ECU sends a set of flows through an output port. Multiple traffic types, like control signals, media signals, diagnostics signals, and infotainment signals, will share the same physical infrastructure. It is inevitable that some types of flows with individual timing requirements will be mapped to the same class [19]. Two SR traffic classes may not be enough to ensure fulfillment of all constraints for all flows with individual timing requirements. Now, some TSN specifications are in progress to guarantee time-critical transmission. On the other hand, for traffic with less-rigid timing requirements, additional SR classes should be considered.

Cao et al. [37] gives a tight bound on the worst-case interference analysis for individual priority classes H (high priority), M (medium priority) and L (BE priority) in an AVB switch. However, it still lacks of the analysis of multiple SR classes. IEEE 802.1Qav [8] has bulit a general formula to determine the worst-case queuing delay experienced by any SR class. When calculating the worst-case queuing delay for an additional SR class with lower priority than class A and B, it looks at higher priority classes (class A and B) together as a single class to perform the analysis and deduction. Thus, the delay result for the additional SR class is pessimistic. The approach proposed in this paper is to reduce the pessimism in the analysis to provide tighter delay bounds for an additional SR class traffic. The primary contributions are summarized as follows:

1. We evaluate the impact of CBS on different priority flows. Particularly, credit variation of class B under constraints of class A at an AVB switching output port is indicated, and the transmission condition for an additional SR class traffic is specified.
2. We identify two types of worst-case queuing delay models for an additional SR class traffic and build a necessary condition for the appearance of each model.
3. We investigate the evolutions of credit A and B for each of the two worst-case queuing delay models and propose an algorithm to determine when the credits of both class A and B are negative. Then, the worst-case queuing delay bounds for an additional SR class are achieved.

### 1.3. Organization

The rest of this paper is organized as follows. Section 2 gives an overview of the Ethernet-AVB network and describes the system model for studying the worst-case queuing delay of an additional SR traffic class. In Section 3, the performance evaluation for the optimization model is implemented. Numerical results are presented in Section 4 for verification our analysis and are followed by our conclusion in Section 5.

## 2. System Model

### 2.1. Context of Ethernet-AVB

Ethernet-AVB [5,6,7,8] comprises some IEEE 802.1 standards for low latency flows. IEEE 802.1 BA [5] defines the Ethernet-AVB system and default configuration. IEEE 802.1AS [6] is specified to ensure the time synchronization requirements for time-sensitive applications, which is based on the IEEE 1588-precision time protocol and provides a synchronization error less than 1 μs over seven hops. IEEE 802.1Qat defines a Stream Reservation Protocol (SRP) [7] to accomplish the reservation request along the path in three steps: stream advertisement, registration and de-registration. According to 802.1Qat, the bandwidth requirement (bits/s units) of a given SR class flow is given by

(1) Bandwidth=(MFS+Overhead)×8×MIF/CMI,

where MFS (Maximum Frame Size) is the maximum frame size of the considered SR class flow. CMI (Class Measurement Interval) is a periodical time interval with 125 μs for class A and 250 μs for class B. In order to expand the application scope, CMI can perhaps be generalised with different values by some switch providers, as assumed in ref. [31]. MIF (Maximum Interval Frame) is the maximum number of frames transmitted during one CMI.

**Figure 1:**
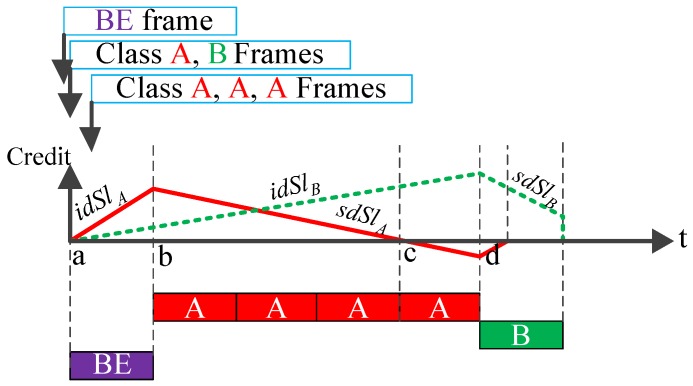
Credit-based shaper process.

We focus on IEEE 802.1Qav [8], which defines queuing and forwarding policy. Each Ethernet-AVB output port imposes a CBS algorithm for each SR class to accomplish traffic shaping. The CBS process is the following, as depicted in Figure 1 (inspired by [8] (Fig L-4)).

- If a SR class frame is waiting for transmission (there is conflicting traffic blocking the output port or the credit is negative), the credit increases at its *idleSlope* (*idSl* for short) rate. *IdSl* represents the maximum guaranteed bandwidth fraction allocated to a given SR class. At most, 75% bandwidth usage is allocated to all SR traffic classes.
- If the corresponding credit allocated to a given SR class is not negative and the link is idle, the transmission of the SR frame is only allowed when there is no higher-priority traffic awaiting transmission or the corresponding higher priority class credit is not enough for transmission. As the transmission proceeds, the credit decreases at the rate of *sendSlope* (*sdSl* for short), and in the worst-case scenario, a maximum-sized frame continues its emission at zero credit up to completion, even if the credit becomes negative. The parameter *sdSl* obeys: sdSl=idSl−linkspeed.
- If there is no further SR frame queued and the current credit is negative, credit will increase to zero at the rate of *idSl*. Otherwise, if there are no frames in the SR class queue and its credit is positive, credit is immediately set to zero.

The delay experienced by a frame in a queue can be decomposed into two parts.

- First, the delay between the instant the frame is enqueued and the instant it becomes the head of queue.
- Second, the delay between becoming first frame of queue and the instant it is selected for emission.

Let X represent an SR traffic class. For the non-preemptive SPQ scheduling mechanism, the queuing delay experienced by the first frame of SR class X queue can be broken out into two components [8]:

- The delay is caused by the frame that was selected for transmission an arbitrarily small time before frame X arrived. In the worst-case scenario, it is the transmission time of a maximum-sized frame with lower priority than frame X’s class.
- The delay is caused by queued-up frames with higher priority than frame X’s class.

As depicted in Figure 1 and from [8] (eq L.38), the queuing delay experienced by the first frame of SR class A queue is given by

(2) $$qDelay_{A}=t_{ab}=M_{0}/R_{0},$$

where $R_{0}$ represents the port transmission rate and $M_{0}$ is the maximum length of an interference frame in the BE class. Let $R_{X}$ denote the *idleSlope* for SR class X and $M_{X}$ the maximum length of a frame in SR class X. Then the *sendSlope* for SR class X is $\left(R_{X}-R_{0}\right)$. In terms of class A, calculating the queuing delay experienced by the first frame of SR class B queue is easy, which is given by

(3) $$\begin{array}{c} qDelay_{B}=t_{ab}+t_{bd}\\ =M_{0}/R_{0}+\left[R_{A}\left(M_{0}/R_{0}\right)-\left(R_{A}-R_{0}\right)\left(M_{A}/R_{0}\right)\right]/\left(R_{0}-R_{A}\right)\\ =M_{0}/\left(R_{0}-R_{A}\right)+M_{A}/R_{0}. \end{array}$$

However, calculating the queuing delay experienced by an additional SR class with lower priority than class A and B is more difficult. When computing the worst-case queuing delay of an additional SR traffic, a trick in 802.1Qav is to looks at higher priority classes together as a single class. By using the sum of the credits available to all higher priority SR classes, a general formula for calculating the worst-case queuing delay experienced by any SR class X is expressed as follows

(4) $$qDelay_{X}=\left(M_{0}+\sum_{k<X}M_{K}\right)/W_{<X},$$

where $W_{<X}=R_{0}-\sum_{K<X}R_{K}$. “<*X*” is a subscript for the sum of all classes with higher priority than class X [8] (eq L.37).

### 2.2. Optimization Model

**Figure 2:**
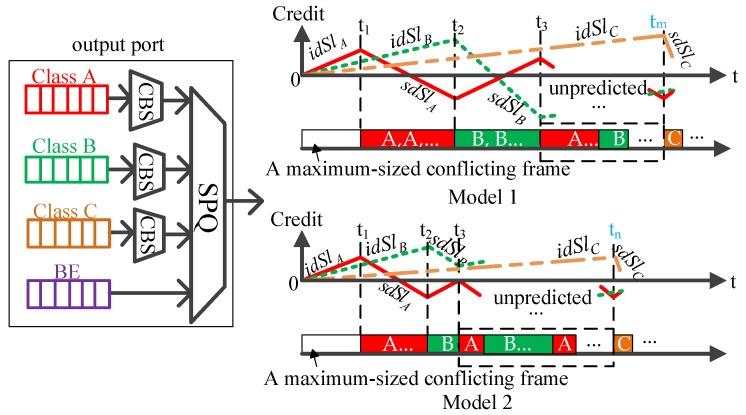
Analytical models (Model 1: Class B frames are forwarded until its credit drains; Model 2: Class B frames stop being forwarded when the credit of class A replenishes to zero).

In this section, we define a worst-case analytical model that we use throughout the paper. The existing two SR classes in the standard Ethernet-AVB network are extended into three SR classes A, B and C, as depicted in Figure 2. Class C has lower priority than class A and B. Denote that N represents the SR traffic class (N∈{A,B,C}) associated with the CBS algorithm and dedicated bandwidth allocation. Flows not belonging to class N are treated as BE flows with the lowest priority. The queue for each SR class is full, and the corresponding credit starts at zero. In ref. [35], it has been proved that for a frame of an AVB SR class, the worst-case scenario can always be found when considering zero initial credit of the corresponding SR class at each output port along the path. Frames belonging to one SR class and the class with higher priority arrive right after a maximum-sized conflicting frame of a lower prioritized class has started to transmit. When the transmission of frames in a given SR class is allowed, the corresponding credit decreases and a maximum-sized frame continues its emission at zero credit up to completion. The credits of the other SR classes increase in this process. The transmission of lower priority SR class frames is only allowed when the credits of all higher priority SR classes are negative.

However, it is difficult to determine the worst-case queuing delay experienced by SR class C, which is affected by class A and B’s flow shaping operation. In addition, the usage of *idSl* and *sdSl* provides great flexibility for Ethernet-AVB flow control. Particularly, if the corresponding *idSl* rate is increased, the transmission speed for SR class flows is accelerated and the credit recovers quickly. Otherwise, if the corresponding *idSl* value is decreased, the transmission speed is slowed down. When calculating the worst-case queuing delay experienced by the first frame of SR class C queue, the key point is to determine the evolution trend of credit A and B.

Class A frames have the highest priority. As depicted in Figure 2, in the worst-case scenario, the transmission of class A frames is blocked by a maximum-sized conflicting frame with lower priority than class A, and class A’s credit increases. After the transmission of the conflicting frame has completed, class A frames start to be transmitted, and credit decreases. Credits of class B and C increase in this process. After the first transmission of class A frames has completed, class A’s credit is not enough for transmission and needs to accumulate. Then, the first transmission of class B frames is allowed and class B’s credit decreases. Meanwhile, credits of class A and C increase. However, the class B frame may stop being forwarded after the credit of class A replenishes to zero. As depicted in Figure 2, during the first transmission of class B frames, whether class B’s credit falls below zero lies in the speed of class A’s credit recovery and will lead to the corresponding Model 1 and Model 2.

Subsequently, some combination of class A and B frames are transmitted after the credit of class A and class B replenishes to zero. As depicted in Figure 2, the subsequent possibilities of frames being transmitted and the subsequent evolution of credit A and B are difficult to predict.

Thus, to determine the worst-case queuing delay experienced by the first frame of SR class C queue, the necessary conditions for the appearance of Model 1 and Model 2 should first be built; then, the evolution trend of credit A and B for each model should be determined. The next section will perform the analysis in detail.

## 3. Performance Evaluation

### 3.1. Necessary Conditions for the Appearance of Model 1 and Model 2

**Figure 3:**
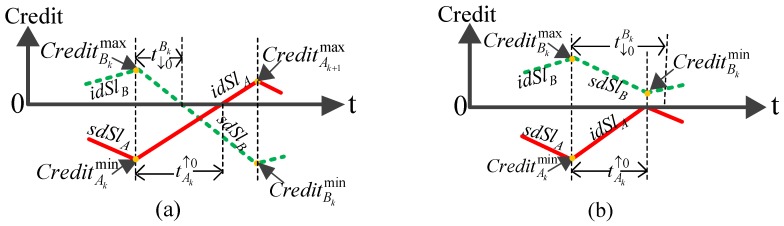
The impact of CBS on class B flows. (**a**) Class B’s credit has fallen to zero before that class A’s credit replenishes to zero; (**b**) Class B’s credit has not fallen to zero before that class A’s credit replenishes to zero.

The following notations are used in the analysis models:

Assume that the queue for each SR class is full. Consider the frame sequence before the emission of the first C frame: it starts with an optional non SR frame, then it is an alternation of sequences of A frames and B frames. For example, if the output link is AAABBABAC, they are 3 sequences of A frames (AAA, A and A) and 2 sequences of B frames (BB, B). Let $Credit_{N_{k}}^{\max}$ ($Credit_{N_{k}}^{\min}$) be the maximal (minimal) bound of the credit during the transmission of the kth sequence of frames of the SR class N (k=1,2,…,n), as depicted in Figure 3.

*R* is the transmission rate of the network. $L_{N}^{\max}$ ($L_{BE}^{\max}$) is the maximum size of a frame of class N (BE class). $L_{lp(N)}^{\max}$ represents the maximum size of a conflicting frame of a lower priority class than class N. Then, the expressions $L_{lp(A)}^{\max}=\max\left\{L_{B}^{\max},L_{C}^{\max},L_{BE}^{\max}\right\}$, $L_{lp(B)}^{\max}=\max\left\{L_{C}^{\max},L_{BE}^{\max}\right\}$ and $L_{lp(C)}^{\max}=\max\left\{L_{BE}^{\max}\right\}$ are achieved. $t_{A_{k}}^{↑0}$ represents the duration when class A’s credit increases from $Credit_{A_{k}}^{\min}$ to zero and $t_{↓0}^{B_{k}}$ represents the duration when class B’s credit decreases from $Credit_{B_{k}}^{\max}$ to zero; then,

(5) $$t_{A_{k}}^{↑0}=-Credit_{A_{k}}^{\min}/idSl_{A},$$

(6) $$t_{↓0}^{B_{k}}=-Credit_{B_{k}}^{\max}/sdSl_{B}.$$

If the transmission of class B frames is allowed and $t_{A_{k}}^{↑0}>t_{↓0}^{B_{k}}$, class B’s credit has fallen to zero before that class A’s credit replenishes to zero and a maximum-sized class B frame can continue to be forwarded at credit zero in the worst-case scenario, as depicted in Figure 3a. Otherwise, if the transmission of class B frames is allowed and $t_{A_{k}}^{↑0}\leq t_{↓0}^{B_{k}}$, class B’s credit cannot continue to decrease at the $sdSl_{B}$ rate at the time when class A’s credit replenishes to zero. It will increase at the $idSl_{B}$ rate until the transmission of a maximum-sized class A frame has been completed, as depicted in Figure 3b.

Thus, when the first transmission of class B is allowed (k=1), the necessary conditions are $t_{A_{1}}^{↑0}>t_{↓0}^{B_{1}}$ for Model 1 and $t_{A_{1}}^{↑0}\leq t_{↓0}^{B_{1}}$ for Model 2, as depicted in Figure 2, where $t_{A_{1}}^{↑0}$ is the duration when class A’s credit increases from $Credit_{A_{1}}^{\min}$ to zero, and $t_{↓0}^{B_{1}}$ is the duration when class B’s credit decreases from $Credit_{B_{1}}^{\max}$ to zero. Next, we discuss how to determine the values of these parameters.

Class A frames have priority over all other traffics. When the transmission is allowed, frames will be transmitted back-to-back without interrupting until the credit drains. In addition, in the worst-case scenario, a maximum-sized frame can still be forwarded when the credit decreases to zero. The check of the minimum credit value is performed at the end of the emission of this maximum-sized frame. So, the value of $Credit_{A_{k}}^{\min}$ is constant, which is given by

(7) $$Credit_{A_{k}}^{\min}=\left(L_{A}^{\max}/R\right)\times sdSl_{A}.$$

$Credit_{A_{1}}^{\max}$ is the amount of credit that can be accumulated during the transmission time of a maximum-sized conflicting frame with lower priority than class A; then,

(8) $$Credit_{A_{1}}^{\max}=\left(L_{lp(A)}^{\max}/R\right)\times idSl_{A}.$$

$Credit_{B_{1}}^{\max}$ is the amount of credit that can be accumulated during the transmission time of a maximum-sized conflicting frame with lower priority than class B plus the transmission time of the maximum numbers of class A frames, which is given by

(9) $$\begin{array}{c} Credit_{B_{1}}^{\max}=idSl_{B}\left(\frac{L_{lp(B)}^{\max}}{R}+\frac{Credit_{A_{1}}^{\max}-Credit_{A_{1}}^{\min}}{-sdSl_{A}}\right)\\ =idSl_{B}\left(\frac{L_{lp(B)}^{\max}}{R}+\frac{L_{A}^{\max}}{R}-\frac{L_{lp(A)}^{\max}}{R}\frac{idSl_{A}}{sdSl_{A}}\right), \end{array}$$

where $Credit_{A_{1}}^{\min}=\left(L_{A}^{\max}/R\right)\times sdSl_{A}$, as seen in (7). Using (5) and (6), the values of $t_{A_{1}}^{↑0}$ and $t_{↓0}^{B_{1}}$ are given by

(10) $$t_{A_{1}}^{↑0}=\frac{-Credit_{A_{1}}^{\min}}{idSl_{A}}=\frac{L_{A}^{\max}}{R}\frac{\left(-sdSl_{A}\right)}{idSl_{A}},$$

and

(11) $$\begin{array}{cc} t_{↓0}^{B_{1}}= & \frac{-Credit_{B_{1}}^{\max}}{sdSl_{B}}\\ = & \frac{idSl_{B}}{\left(-sdSl_{B}\right)}\;\left(\frac{L_{lp(B)}^{\max}}{R}+\frac{L_{A}^{\max}}{R}-\frac{L_{lp(A)}^{\max}}{R}\frac{idSl_{A}}{sdSl_{A}}\right). \end{array}$$

As seen in (10) and (11), the effect of CBS depends on flow loads and *idleSlope* configurations. Using this configuration information, the values of $t_{A_{1}}^{↑0}$ and $t_{↓0}^{B_{1}}$ can be calculated and compared. Then, the worst-case queuing delay model of class C is determined.

### 3.2. Analysis of the Evolution of Credit for Two Models

**Figure 4:**
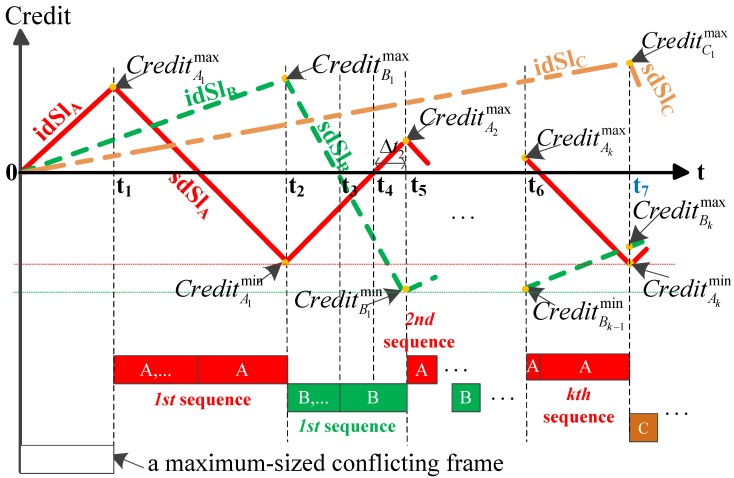
Credit evolution for Model 1.

**Theorem** **1.** *(Credit evolution analysis for Model 1) If $t_{A_{1}}^{↑0}>t_{↓0}^{B_{1}}$, the maximum values of credit A and B ($Credit_{A_{k}}^{\max}$ and $Credit_{B_{k}}^{\max}$, respectively) become smaller and smaller as the value of k increases, and class B’s credit will drain during every transmission of class B frames, as depicted in Figure 4. The following relationships are achieved: $Credit_{A_{k+1}}^{\max}<Credit_{A_{k}}^{\max}$, $Credit_{B_{k+1}}^{\max}<Credit_{B_{k}}^{\max}$ and $Credit_{B_{k}}^{\min}=\left(L_{B}^{\max}/R\right)\times sdSl_{B}$.*

**Proof** **of** **Theorem** **1.** Since $t_{A_{1}}^{↑0}>t_{↓0}^{B_{1}}$, $Credit_{B_{1}}^{\min}=\left(L_{B}^{\max}/R\right)\times sdSl_{B}$ is obtained according to the previous analysis. Let $\Delta t_{2}$ be the duration when class A’s credit increases from zero to $Credit_{A_{2}}^{\max}$ (see [$t_{4}$,$t_{5}$] in Figure 4), so $Credit_{A_{2}}^{\max}=\Delta t_{2}\times idSl_{A}$. Then, it is possible to express the relationship: $\Delta t_{2}<(L_{B}^{\max}/R)$ with $t_{A_{1}}^{↑0}>t_{↓0}^{B_{1}}$ assumed. Please note that the maximum size of a conflicting frame for class A is $L_{lp(A)}^{\max}=\max\left\{L_{B}^{\max},L_{C}^{\max},L_{BE}^{\max}\right\}$; comparing the value of $Credit_{A_{2}}^{\max}$ with the value of $Credit_{A_{1}}^{\max}$ seen in (8), the following relationship is achieved:

(12) $$Credit_{A_{2}}^{\max}<Credit_{A_{1}}^{\max}.$$

Class B’s credit reaches the value of $Credit_{B_{k+1}}^{\max}$ when class A’s credit reaches the value of $Credit_{A_{k+1}}^{\min}$. The value of $Credit_{B_{k+1}}^{\max}$ is computed by the following equation:

(13) $$Credit_{B_{k+1}}^{\max}=idSl_{B}\left(\frac{Credit_{A_{k+1}}^{\max}-Credit_{A_{k+1}}^{\min}}{-sdSl_{A}}\right)+Credit_{B_{k}}^{\min},$$

where $Credit_{A_{k+1}}^{\min}=\left(L_{A}^{\max}/R\right)\times sdSl_{A}$ and $Credit_{B_{1}}^{\min}=\left(L_{B}^{\max}/R\right)\times sdSl_{B}$. Comparing the value of $Credit_{B_{2}}^{\max}$ computed from (13) with the value of $Credit_{B_{1}}^{\max}$ seen in (9), we have

(14) $$Credit_{B_{2}}^{\max}<Credit_{B_{1}}^{\max}.$$

Thus, the relationship $t_{↓0}^{B_{2}}<t_{↓0}^{B_{1}}<t_{A_{1}}^{↑0}$ is deduced. In addition, since the value of $Credit_{A_{k}}^{\min}$ is constant, $t_{A_{1}}^{↑0}=t_{A_{2}}^{↑0}$ is obtained by using (5). Then, $t_{↓0}^{B_{2}}<t_{A_{2}}^{↑0}$ and $Credit_{B_{2}}^{\min}=\left(L_{B}^{\max}/R\right)\times sdSl_{B}$ are achieved. Furthermore, class A’s credit reaches the value of $Credit_{A_{k+1}}^{\max}$ when class B’s credit reaches the value of $Credit_{B_{k}}^{\min}$. The value of $Credit_{A_{k+1}}^{\max}$ is computed by the following equation:

(15) $$Credit_{A_{k+1}}^{\max}=idSl_{A}\left(\frac{Credit_{B_{k}}^{\max}-Credit_{B_{k}}^{\min}}{-sdSl_{B}}\right)+Credit_{A_{k}}^{\min}.$$

Using (12) and (14), (13) and (15) continue to be computed iteratively. Then, we have: $Credit_{A_{k+1}}^{\max}<Credit_{A_{k}}^{\max}$ and $Credit_{B_{k+1}}^{\max}<Credit_{B_{k}}^{\max}$. The relationship $t_{A_{k}}^{↑0}>t_{↓0}^{B_{1}}>t_{↓0}^{B_{2}}>\ldots >t_{↓0}^{B_{k}}$ is derived. Finally, $Credit_{B_{k}}^{\min}=\left(L_{B}^{\max}/R\right)\times sdSl_{B}$ is obtained. ☐

**Figure 5:**
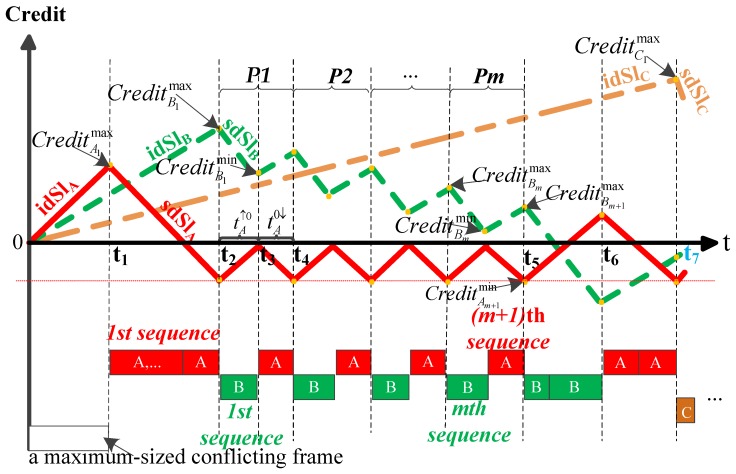
Credit evolution for Model 2.

**Theorem** **2.** *(Credit evolution analysis for Model 2) If $t_{A_{1}}^{↑0}\leq t_{↓0}^{B_{1}}$, during the first or first few times of class B transmission, the minimum value of credit B is not less than zero (see [$t_{2}$,$t_{5}$] in Figure 5). However, subsequently, credit evolution will follow what was analyzed in Model 1 (see [$t_{5}$,$t_{7}$] in Figure 5). The following relationships are achieved: $Credit_{B_{k+1}}^{\max}<Credit_{B_{k}}^{\max}$, and $Credit_{A_{i}}^{\max}>0\Rightarrow Credit_{A_{i}}^{\max}<\inf\left\{Credit_{A_{j}}^{\max}\left|\;j<i,\;\right.Credit_{A_{j}}^{\max}>0\right\}$.*

**Proof** **of** **Theorem** **2.** During the first transmission of class B, if $t_{A_{1}}^{↑0}\leq t_{↓0}^{B_{1}}$, class B frames cannot continue to be forwarded at the time when class A’s credit replenishes to zero (see $t_{3}$ in Figure 5). Define that *P* is a periodical time interval that is characterized by the duration when class A’s credit increases from $Credit_{A_{k}}^{\min}$ to zero plus the duration when the class A’s credit decreases from zero to $Credit_{A_{k+1}}^{\min}$ (see [$t_{2}$,$t_{4}$] in Figure 5). During a period of *P*, let $t_{B}^{↓}$ represent the duration when class B’s credit decreases from $Credit_{B_{k}}^{\max}$ to $Credit_{B_{k}}^{\min}$, and let $t_{B}^{↑}$ represent the duration when the class B’s credit increases from $Credit_{B_{k}}^{\min}$ to $Credit_{B_{k+1}}^{\max}$, which are given by

(16) $$\begin{array}{c} t_{B}^{↓}=t_{A}^{↑0}=-Credit_{A_{k}}^{\min}/idSl_{A}, \end{array}$$

(17) $$\begin{array}{c} t_{B}^{↑}=t_{A}^{0↓}=Credit_{A_{k+1}}^{\min}/sdSl_{A}, \end{array}$$

where $t_{A}^{↑0}$ is the duration when class A’s credit increases from $Credit_{A_{k}}^{\min}$ to zero and $t_{A}^{0↓}$ is the duration when class A’s credit decreases from zero to $Credit_{A_{k+1}}^{\min}$.

Define that $Credit_{B}^{↓}$ and $Credit_{B}^{↑}$ are the credit variation during the duration $t_{B}^{↓}$ and $t_{B}^{↑}$ respectively; then,

(18) $$\begin{array}{c} Credit_{B}^{↓}=\frac{Credit_{A_{k}}^{\min}}{idSl_{A}}\times sdSl_{B}, \end{array}$$

(19) $$\begin{array}{c} Credit_{B}^{↑}=\frac{Credit_{A_{k+1}}^{\min}}{sdSl_{A}}\times idSl_{B}. \end{array}$$

According to 802.1Qat, assume that $idSl_{A}+idSl_{B}=S\leq 75\%\times R$; then

(20) $$\begin{array}{c} \frac{-sdSl_{B}}{idSl_{A}}=\frac{\left(R-S\right)+idSl_{A}}{idSl_{A}}>1, \end{array}$$

(21) $$\begin{array}{c} \frac{idSl_{B}}{-sdSl_{A}}=\frac{idSl_{B}}{\left(R-S\right)+idSl_{B}}<1. \end{array}$$

Hence, the expression $Credit_{B}^{↓}>Credit_{B}^{↑}$ is obtained. After the number of *m* (m=1,2,…,m<k) periods of *P* (see [$t_{2},t_{5}$] in Figure 5) only if *m* meets the following relationship:

(22) $$\begin{array}{c} Credit_{B}^{↑}\leq Credit_{B_{1}}^{\max}-m\left(Credit_{B}^{↓}-Credit_{B}^{↑}\right)<Credit_{B}^{↓}, \end{array}$$

the value of $Credit_{B_{m+1}}^{\max}$ is small enough to satisfy: $t_{A_{m+1}}^{↑0}>t_{↓0}^{B_{m+1}}$ (see [$t_{5},t_{6}$] in Figure 5) and computed by

(23) $$\begin{array}{c} Credit_{B_{m+1}}^{\max}=Credit_{B_{1}}^{\max}-m\left(Credit_{B}^{↓}-Credit_{B}^{↑}\right). \end{array}$$

From then on (see $t_{5}$ in Figure 5), during the transmission of class B frames, class B’s credit value will fall below zero. The subsequent credit evolution trend is just as described in Theorem 1. Thus, we have: $Credit_{B_{k+1}}^{\max}<Credit_{B_{k}}^{\max}$ and $Credit_{A_{i}}^{\max}>0\Rightarrow Credit_{A_{i}}^{\max}<\inf\left\{Credit_{A_{j}}^{\max}\left|\;j<i,\;\right.Credit_{A_{j}}^{\max}>0\right\}$. ☐

### 3.3. Determining the Worst-Case Queuing Delay Experienced by the First Frame of SR Class C Queue

As in the previous analysis, when the CBS configurations are given, the worst case queuing delay model is determined. According to the discussion on credit evolution for the two models depicted in Theorems 1 and 2, the values of credit A and B become smaller and smaller except that the credit of class A is bigger at one moment in Model 2 (see $t_{6}$ in Figure 5). Finally, both credits of class A and B are negative, and then the transmission of class C frames is allowed. In addition, the worst-case queuing delay experienced by the first frame of SR class C queue is obtained. Using Theorems 1 and 2, the detailed algorithm steps of the iterative method are presented in Algorithm 1.

**Algorithm 1** The worst-case queuing delay experienced by the first frame of SR class C queue**Input:***R*, $idSl_{N}$, $L_{N}^{\max}$ and $L_{BE}^{\max}$ **Output:** The worst-case queuing delay experienced by the first frame of SR class C queue: $T_{C}$
1:Initialize the model parameters: $Credit_{A_{1}}^{\max}$, $Credit_{B_{1}}^{\max}$, $Credit_{A_{k}}^{\min}$, $t_{A_{1}}^{↑0}$, $t_{↓0}^{B_{1}}$, $Credit_{B}^{↓}$, $Credit_{B}^{↑}$
2:**if** ($t_{A_{1}}^{↑0}\leq t_{↓0}^{B_{1}}$) **then**3: **repeat**4:  $Credit_{B_{k+1}}^{\max}\leftarrow \left(Credit_{B_{k}}^{\max}-Credit_{B}^{↓}+Credit_{B}^{↑}\right)$5: **until** ($Credit_{B_{k}}^{\max}<Credit_{B}^{↓}$)6:**end if**7:**repeat**8: $Credit_{A_{k+1}}^{\max}$$\leftarrow (idSl_{A}\frac{Credit_{B_{k}}^{\max}-L_{B}^{\max}/R\times sdSl_{B}}{-sdSl_{B}}+\frac{L_{A}^{\max}}{R}sdSl_{A})$9: **if** ($Credit_{A_{k+1}}^{\max}\geq 0$) **then**10:  $Credit_{B_{k+1}}^{\max}$$\leftarrow (idSl_{B}\frac{Credit_{A_{k+1}}^{\max}-L_{A}^{\max}/R\times sdSl_{A}}{-sdSl_{A}}+\frac{L_{B}^{\max}}{R}sdSl_{B})$11: **else**12:  break;13: **end if**14:**until** ($Credit_{B_{k+1}}^{\max}<0$)15:**return**$T_{C}$


## 4. Numerical Results

### 4.1. Simple Cases Illustration

First, two simple cases are studied to verify the correctness and advantage of our models. Four flows assigned with class A, B, C and BE are forwarded at an Ethernet-AVB switch output port. The transmission rate of the network is 100 Mbits/s. The CBS configurations are given in Table 1.

**Figure 6:**
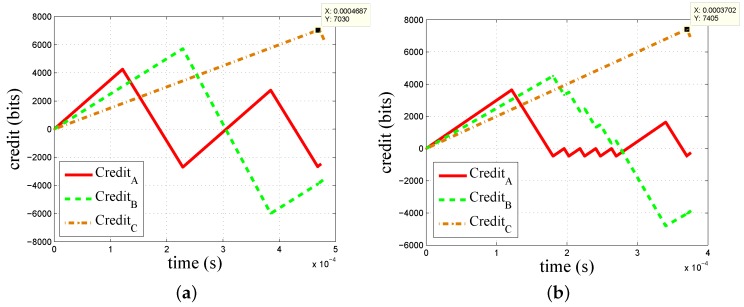
The worst-case queuing delay results. (**a**) Results for Case 1. (**b**) Results for Case 2.

The results for Case 1 are shown in Figure 6a. Using (5) and (6), the expressions $t_{A_{1}}^{↑0}=77$ μs and $t_{↓0}^{B_{1}}=48$ μs are obtained. The evolution of credit is consistent with what is described in Theorem 1 with $t_{A_{1}}^{↑0}>t_{↓0}^{B_{1}}$. The results for Case 2 are shown in Figure 6b. Using (5) and (6), the expressions $t_{A_{1}}^{↑0}=16$ μs and $t_{↓0}^{B_{1}}=56$ μs are obtained. The evolution of credit is consistent with what is described in Theorem 2 with $t_{A_{1}}^{↑0}<t_{↓0}^{B_{1}}$.

The comparison results of a worst-case queuing delay between our method and Formulas (2)–(4) mentioned in 802.1Qav [8] are shown in Table 2.

For class A and B, the worst-case queuing delay obtained from our models are the same as those obtained by the 802.1Qav standard. The correctness of our models is verified. For class C, the results show that our method brings significant improvement of up to 23% in Case 1 and 13% in Case 2. The advantage of our models is illustrated.

### 4.2. Evaluation with Different Size and IdleSlope

**Figure 7:**
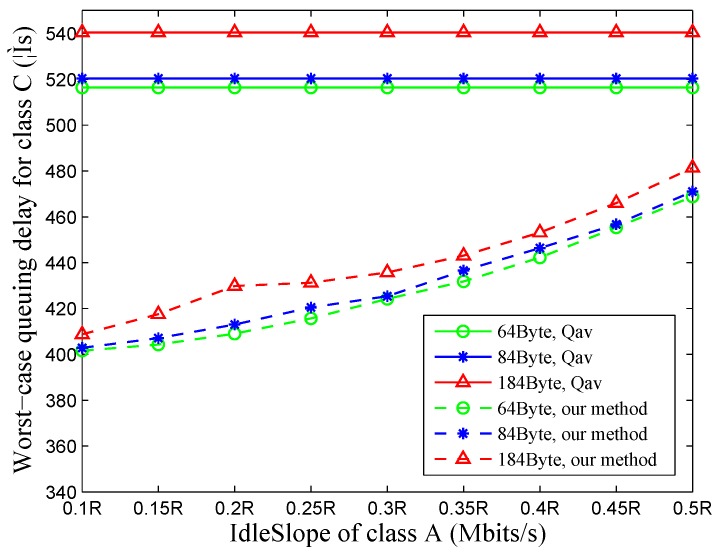
Influence of frame lengths and *idleSlope*.

In this section, the influence of frame lengths and the *idleSlope* of class A and B on the worst-case queuing delay of class C is investigated. Assume that the value of $idSl_{C}$ is 0.15R Mbits/s (R=100 Mbits/s); then, the sum value of $idSl_{A}$ and $idSl_{B}$ is 0.6R Mbits/s. Let the value of $idSl_{A}$ increase from 0.1R Mbits/s to 0.5R Mbits/s by 0.05R; then, the value of $idSl_{B}$ decreases from 0.5R Mbits/s to 0.1R Mbits/s by 0.05R correspondingly. Frame lengths of class B, C and BE are 1000 Byte, 1518 Byte and 1518 Byte, respectively. Small-sized frames are assigned to class A, and the sizes of 64 Byte, 84 Byte and 184 Byte are taken as a reference. As depicted in Figure 7, the results show that our approach produces tighter queuing delay bounds in the case of class C and is capable of reflecting the delay variation over the increasing of frame lengths and the *idleSlope* of higher priority traffic classes.

### 4.3. End-to-End Delay Evaluation

**Figure 8:**
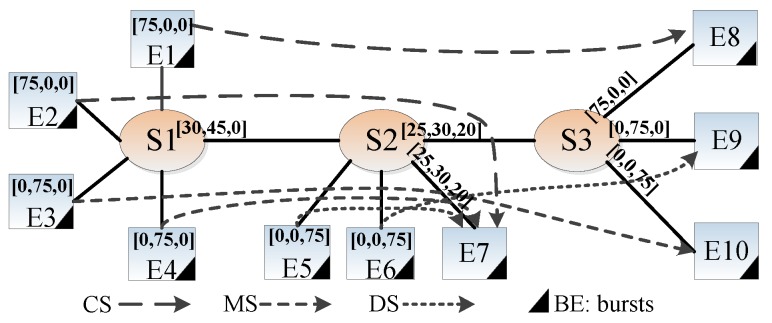
Ethernet-AVB case with Control Signal (CS), Media Signal (MS), Diagnostics Signal (DS) and BE signal and *idleslope* at each port [$idSl_{A}$, $idSl_{B}$, $idSl_{C}$].

In addition, a case study on the worst-case end-to-end delay, which is inspired by [38], is depicted in Figure 8. Ten end systems, E1∼E10, are connected to three Ethernet-AVB switches: S1∼S3. Four types of streams mapped to class A, B, C and BE are transmitted over the network. Control signals (CS) have the highest priority to map into class A streams; media signals (MS) and some diagnostics signals (DS) with less rigid timing requirements are mapped to class B and class C, respectively; background signals are regarded as BE streams, which broadcast in the network.The transmission rate of the network is 100 Mbits/s, and the technology switching delay of each switch is 16 μs. The detailed configurations are shown in Table 3.

**Figure 9:**
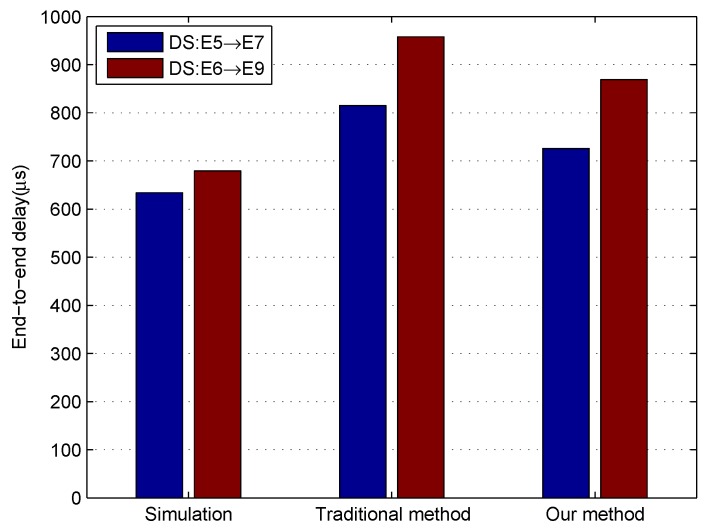
Comparison results on end-to-end delay of class C (Simulation is to use the OMNeT++ simulator to get the maximum observed end-to-end delays. The traditional method and improved method are both employed to use the network calculus approach to obtain the end-to-end delay bounds, in which the service latency of the service curve is calculated by 802.1Qav [8] and our models described in the previous section).

Before Section 4.3, an approach for determining worst-case queuing delay experienced by the first frame of SR class C queue is proposed. In this section, network calculus approach is used to obtain the worst-case end-to-end delay for a class C flow. The network calculus approach has been applied to the switched Ethernet network to guarantee real-time communication even if it provides delay upper bounds with pessimistic computation that the simulation method cannot calculate [23]. The basic concept of network calculus can be found in Appendix A. A flow in class N has a maximum frame size and a class measurement interval. Its arrive curve obeys the leaky bucket model [25]. A typical service curve is the rate-latency function [25]. The worst-case queuing delay calculated by our models and 802.1Qav [8] is regarded as the service latency of the service curve. The service curve is built and proved by Lemma A1 in Appendix A. Then, the flow delay bound is the horizontal deviation between the corresponding arrive curve and service curve. The results show that our method gives tighter end-to-end delay bounds. In addition, we use the OMNeT++ simulator to obtain the maximum observed end-to-end delays as a reference, as depicted in Figure 9.

## 5. Conclusions

In this paper, the worst-case upper-bounded delay for an additional SR traffic class has been investigated. First, the impact of CBS on different priority flows is researched in detail. Second, according to the CBS configurations, two types of worst-case queuing delay models for the additional SR traffic class are identified, and the necessary conditions for each type of model is built. Based on the analytical results, the worst-case execution time required for flows mapped into an additional SR class may be satisfied with appropriate configuration suggestions on the premise of ensuring timing requirements for higher priority SR flows. Furthermore, the credit evolution of each model is analyzed, and an algorithm for calculating the worst-case queuing delay is given. Compared with the method mentioned in 802.1Qav, our method brings a significant improvement of up to 22.9% in Case 1 and 13.3% in Case 2. Finally, our approach reduces the pessimism to provide tight end-to-end delay bounds by using network calculus. In addition, it is important to note that our approach can be extended to an arbitrary number of SR classes. It is expected that our approach may guide the design of Ethernet-AVB for in-vehicle networking. The impact of time-critical class CDT traffic on our models will be evaluated in the future.

## Figures and Tables

**Table 1:** CBS configurations.

Case	Class	MaximumFrameSize (Byte)	idleSlope (Mbits/s)
	A	520	35
1	B	1000	25
	C	1518	15
	BE	1518	25
	A	84	30
2	B	800	25
	C	1200	20
	BE	1518	25

**Table 2:** Queuing delay comparison. The ratio shows factor of improvement.

Case	Class	By Qav [μs]	By Our Method [μs]	Ratio
	A	121	121	1:1
1	B	228	228	1:1
	C	608	469	1:0.77
	A	121	121	1:1
2	B	180	180	1:1
	C	427	370	1:0.87

**Table 3:** Configurations.

Traffic Type	Bandwidth (Mbits/s)	Payload (Byte)	CMI (ms)	Class (Priority)
CS	0.672	84	1	A
MS	8.0	1200	1.2	B
DS	4.0	500	1	C
BE	Bursts	1518	Bursts	BE

**Figure A1:**
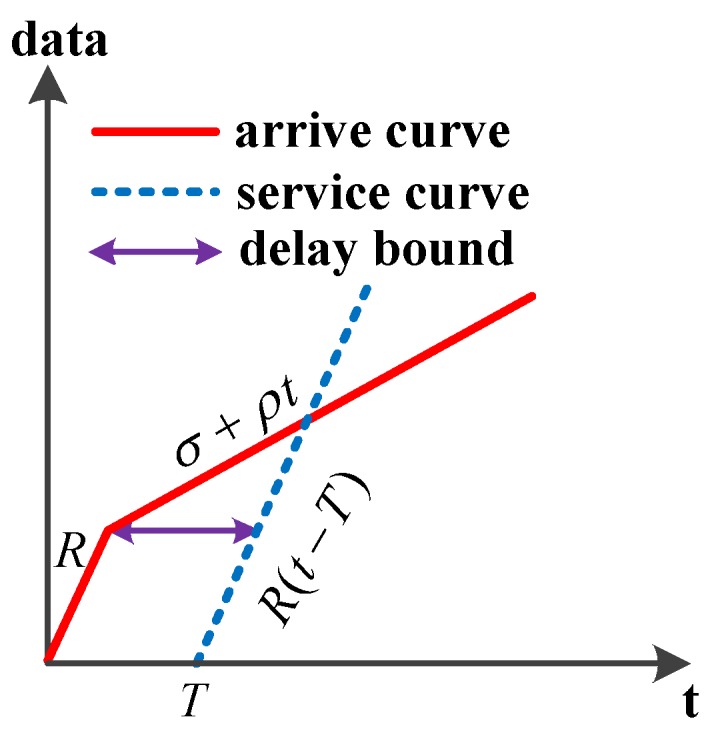
Illustration of the arrive curve and the service curve.

## Appendix A. Network Calculus Approach

Specify that R(t) ($R^{*}\left(t\right)$) is the input (output) function of data flows through a network element; then, R(t) is constrained by the arrive curve α(t) [25] if and only if for all s≤t, R(t)−R(s)≤α(t−s). A typical arrive curve is the leaky bucket function, which is given by

(A1) $$\alpha_{\sigma ,\rho }\left(t\right)=\left\{\begin{array}{c} \sigma +\rho t\;\;\;\;if\;t>0\;\;\;\\ 0\;\;\;\;\;\;\;\;\;\;\;otherwise, \end{array}\right.$$

where σ is the maximum burst tolerance and ρ is the long-term constant rate. If the flow is transmitted through a link with the rate *R*, a tight arrive curve is defined by $\alpha \left(t\right)=\min\{R\times t,\alpha_{\sigma ,\rho }\left(t\right)\}$, as depicted in Figure A1. For a flow belonging to class *N* in the Ethernet-AVB network, $\sigma =L_{N}^{\max}\times MIF\times \left(1-\rho /R\right)$ and $\rho =L_{N}^{\max}\times MIF/CMI$ [32].

A network element offers a flow with R(t) and $R^{*}\left(t\right)$ a service curve β(t)[25] if and only if for all t≥0, $R^{*}\left(t\right)\geq \underset{s\leq t}{\inf}\;\left(R\left(s\right)+\beta \left(t-s\right)\right)$. A typical service curve is the rate-latency function, which is given by

(A2) $$\beta_{R,T}\left(t\right)=R{\left(t-T\right)}^{+}=\left\{\begin{array}{c} R(t-T)\;\;\;\;\;if\;t>0\;\;\;\\ 0\;\;\;\;\;\;\;\;\;\;\;\;\;\;\;otherwise, \end{array}\right.$$

where *R* and *T* represent the service rate and the service latency, respectively.

**Lemma** **A1.** *For a flow belonging to class* N *in the Ethernet-AVB network, if the AVB shaper is allocated with parameters $idSl_{N}$ (the idleSlope of class N) and $T_{N}$ (the worst-case queuing delay experienced by the first frame of SR class N queue) at an output port, the service curve of class N can be defined as $\beta_{N}^{}\left(t\right)=idSl_{N}{\left[t-T_{N}\right]}^{+}$.*

**Proof.** SR class N’s credit is limited by a higher and lower bound. The lower bound is reached when a maximum-sized frame continues its transmission at zero credit up to completion. The credit at the end of the transmission is $sdSl_{N}\left(L_{N}^{\max}/R\right)$. For class A and B, the higher bound of the credit is reached after the first sequence (Theorems 1 and 2), which is given by

(A3) $$Credit_{A}^{\max}=Credit_{A_{1}}^{\max}=idSl_{A}\times T_{A},$$

(A4) $$Credit_{B}^{\max}=Credit_{B_{1}}^{\max}=idSl_{B}\times T_{B}.$$

For class C, the relationship $Credit_{C_{1}}^{\max}=idSl_{C}\times T_{C}$ can be achieved from Theorems 1 and 2. SR class C’s credit accumulates up to the higher bound at the rate of $idSl_{C}$ during the transmission time of the maximum numbers of conflicting frames including a non SR frame and an alternation of sequences of A frames and B frames. Assume that $Credit_{C}^{\max}\neq Credit_{C_{1}}^{\max}$, then it can be deduced that $T_{C}$ is not the worst-case queuing delay of the first frame of SR class C queue, which contradicts the definition of worst-case scenario of Model 1 and Model 2. Thus, we have

(A5) $$Credit_{C}^{\max}=Credit_{C_{1}}^{\max}=idSl_{C}\times T_{C}.$$

So the higher bound is the amount of credit that is accumulated during its worst-case queuing delay time, and it is equal to $\left(idSl_{N}\times T_{N}\right)$. Then in the worst-case scenario, the credit bounds of class *N* is expressed as follows:

(A6) $$\begin{array}{c} sdSl_{N}\left(L_{N}^{\max}/R\right)\leq Credit_{N}\leq (idSl_{N}\times T_{N}). \end{array}$$

Credit variation of a SR class during Δt has been built and proved in ref. [32], and it is given by

(A7) $$\begin{array}{c} \Delta Credit_{N}=idSl_{N}\Delta t-\left[N^{\prime }\left(t+\Delta t\right)-N^{\prime }\left(t\right)\right], \end{array}$$

where $N^{\prime }\left(t\right)$ is the output data flow. Assume that $R_{N}\left(t\right)$ and $R_{N}^{*}\left(t\right)$ are the input and output cumulative function of flows belonging to Class N, and *s* represents the initial congestion time of a switch port, then $Credit_{N}\left(s\right)=0$ and $R_{N}^{*}\left(s\right)=R_{N}\left(s\right)$. Using (A6) and (A7), considering the higher bound of the credit, the credit variation after a period (t−s) is given by

(A8) $$\begin{array}{c} Credit_{N}\left(t\right)-Credit_{N}\left(s\right)=idSl_{N}\left(t-s\right)-\left[R_{N}^{*}\left(t\right)-R_{N}^{*}\left(s\right)\right]\leq \left(idSl_{N}\times T_{N}\right), \end{array}$$

then the last relationship is modified:

(A9) $$\begin{array}{c} R_{N}^{*}\left(t\right)\geq R_{N}^{}\left(s\right)+idSl_{N}\left[\left(t-s\right)-T_{N}\right]\\ \;\;\;\;\;\;\;\;\;=R_{N}^{}\left(s\right)+\beta_{N}^{}\left(t-s\right)\geq \underset{0\leq s\leq t}{\inf}\;\left\{R_{N}^{}\left(s\right)+\beta_{N}^{}\left(t-s\right)\right\}. \end{array}$$

☐

If a flow is constrained by α(t) through a network element offering β(t), the delay bound h(α,β) is the horizontal deviation between α(t) and β(t) [25], which is given by

(A10) $$\begin{array}{c} h\left(\alpha ,\beta \right)=\underset{s\geq 0}{\sup}\;\left(\inf\left\{\tau :\tau \geq 0\;and\;\alpha (s)\leq \beta (s+\tau )\right\}\right). \end{array}$$

In addition, $\alpha^{*}$ constrains the output flow of the given node and is the arrive curve of the input flow of the next node, which is given by

(A11) $$\begin{array}{c} \alpha^{*}=\underset{u\geq 0}{\sup}\;\left\{\alpha \left(t+u\right)-\beta \left(u\right)\right\}. \end{array}$$

Then, the worst-case end-to-end delay of a considered flow is the sum of all delay bounds in network elements along its transmission path.
